# Clinical frailty scale as a predictor of outcome in elderly patients affected by moderate or severe traumatic brain injury

**DOI:** 10.3389/fneur.2023.1021020

**Published:** 2023-04-06

**Authors:** Lucia Zacchetti, Luca Longhi, Rosalia Zangari, Silvia Aresi, Federica Marchesi, Paolo Gritti, Francesco Biroli, Luca Ferdinando Lorini

**Affiliations:** ^1^Department of Anesthesia, Emergency and Critical Care Medicine, Papa Giovanni XXIII Hospital, Bergamo, Italy; ^2^Fondazione per la Ricerca Ospedale di Bergamo (FROM), Papa Giovanni XXIII Hospital, Bergamo, Italy; ^3^Department of Medicine and Surgery, University of Milano-Bicocca, Milan, Italy

**Keywords:** traumatic brain injury, frailty, geriatric, clinical frailty scale, older people

## Abstract

**Background:**

Older age is a well-known risk factor for unfavorable outcome in traumatic brain injury (TBI). However, many older people with TBI respond well to aggressive treatments, suggesting that chronological age and TBI severity alone may be inadequate prognostic markers. Frailty is an age-related homeostatic imbalance of loss of physiologic and cognitive reserve resulting in both limitation in autonomy of activities of daily living and vulnerability to adverse events. We hypothesized that frailty would be associated with 6-month adverse functional outcome in older people affected by moderate or severe TBI.

**Methods:**

This was a single-center prospective observational study. We enrolled consecutive patients aged ≥65 years after TBI with Glasgow Coma Scale ≤13 and admitted to our Neurosurgical Intensive Care Unit. Frailty was evaluated by Clinical Frailty Scale (CFS). Relationships between TBI severity, frailty and extended Glasgow Outcome Scale (GOSE) at 6-month were evaluated.

**Results:**

Sixty patients were studied, 65% were males, their age was 76 years (IQR 70–80) and their admission GCS was 8 (IQR 6–11) with a GCS motor score of 5 (IQR 4–5). Twenty eight were vulnerable-frail (defined as CFS ≥ 4). Vulnerable-frail patients showed greater 6-month mortality and unfavorable outcome compared to non-frail [87% vs. 30% OR and 95% CI: 15.7 (3.9–55.2), *p* < 0.0001 and 92% vs. 51% OR and 95% CI: 9.9 (2.1–46.3), *p* = 0.002]. In univariate analysis patients with unfavorable outcome were more frequently male and vulnerable-frail, had a higher prevalence of pre-existing neurodegenerative disease, abnormal pupil, lower GCS and had worst CT scan characteristics. At multivariate analysis, only CFS ≥ 4 and traumatic subarachnoid hemorrhage remained associated to 6-month outcome.

**Conclusion:**

Frailty was associated with 6 month-outcome, suggesting that the pre-injury functional status could represent an additional indicator to stratify patient’s severity and to predict outcome.

## Background

Over the last decades the epidemiology of traumatic brain injury (TBI) has changed with an increased incidence in older people. The main mechanism of TBI in this population is low velocity falls (1). The collaborative European Neurotrauma Effectiveness Research (CENTER-TBI), a multicenter European-based cohort study of over 4,500 patients, found that 28% of the population was older than 65 years, compared with around 10% in older studies (2). Higher residual disability and mortality among older versus younger individuals with TBI may contribute to the assumption of futility about aggressive management of geriatric TBI. However, many elderly with TBI respond well to aggressive treatments and rehabilitation, suggesting that chronological age and TBI severity assessed with the Glasgow coma scale (GCS) may be inadequate prognostic indicators in this cohort (3).

Increasing attention has been focused on pre-injury functional status, as a possible further prognostic factor. Frailty is an age-related homeostatic imbalance of loss of physiologic and cognitive reserve resulting in both limitations in the autonomy of activities of daily living and vulnerability to adverse events such as diseases. Adverse outcomes associated with frailty are complications, reduced functional outcome and mortality. To date frailty is evaluated using different methods. There is a method based on a “deficit accumulation model,” that evaluates comorbidities, medications, laboratory abnormalities providing a frailty index (FI); a person is considered frail when the FI is greater than 0.2. There is a phenotypic model that evaluates 5 items: unintentional weight loss, strength, physical activity, speed velocity and fatigability; a person is frail when 3 out of 5 items are present. There is a model based on a multidimensional biopsychosocial model, which combines physical and psychosocial domains (4).

The Clinical Frailty Scale is a clinical judgment-based frailty tool that evaluates specific domains including comorbidity, function, and cognition to generate a score ranging from 1 (very fit) to 9 (terminally ill) (5, 6). The CSF has been shown to be an adequate tool to evaluate frailty at ICU admission and recently it has been shown to be associated with short-term mortality in older people admitted to ICU (7). In the setting of TBI frailty evaluated with either the FI or a modified 5-item FI has been proven to be independently associated with mortality and unfavorable functional outcome in patients with TBI of all ages (8, 9). However the classic FI requires the physicians to consider a list of several disorders (10) and may be difficult to incorporate into a busy clinical practice. On the contrary, CSF is readily available at the bedside, easy to understand and is an optimal tool to use on admission to the ICU.

We hypothesized that frailty, measured by CFS, would be associated with 6-month adverse functional outcome in older people affected by TBI. The aim of this preliminary study was to evaluate the association between frailty and GOSE at 6-months in older patients after TBI.

## Materials and methods

This was a preliminary prospective observational study conducted at the Ospedale Papa Giovanni XXIII, Begamo Italy. This study was conducted in accordance with the Declaration of Helsinki and approved by the local Ethics Board on December 10, 2016 (identification number NCT03810222). Inclusion criteria: consecutive patients aged ≥65 years admitted to the Neurosurgical Intensive Care Unit (NICU) of our hospital between 1st of January 2017 and 31th of May 2017, after a diagnosis of moderate–severe TBI [Glasgow Coma Scale (GCS) ≤ 13].

Basic principles of patients’ management were: surgical evacuation of hematomas causing mass effect, monitoring of intracranial pressure in comatose patients with positive CT scan, treatment of intracranial hypertension starting at a threshold of 20 mmHg, maintenance of cerebral perfusion pressure greater than 60 mmHg and avoidance of systemic insults known to be detrimental for the injured brain (11).

The following data were collected at NICU admission: demographic characteristics; occurrence of hypotension and hypoxia defined, respectively, as systolic arterial pressure ≤ 90 mmHg, and SpO2 < 90% or pO2 < 60 mmHg; post-resuscitation GCS (total and motor score); pupils’ reactivity (dichotomized in “both reactive” vs. “one or both dilated or fixed”). Three CT scans of the brain were routinely taken in the first 24 h and further CT scans were repeated in case of neuroworsening/intracranial hypertension or for other reasons at the discretion of physicians. The status of basal cisterns, midline shift, traumatic subarachnoid hemorrhage was evaluated in the first and worst CT scan. The outcome was evaluated at 6 months using the extended Glasgow Outcome Scale (GOSE) and was dichotomized in unfavorable (score 1–3) and favorable (score 4–8) outcomes. The choice to consider GOSE = 4 as a good outcome was decided “*a priori*” before data collection, because frail older people could be already dependent on others for daily activities, therefore resuming relative independence at home could be considered a realistic favorable outcome.

Two trained physicians (LZ and SA) submitted separately the CFS to the patient’s next of kin. In case of different CSF score collected for the same patient, a third senior physician (LL) was asked to resolve the disagreement. CFS is a 9-point scale, with a score of 1 indicating an energic and well-motivated person who exercises regularly and is in the fittest group for her/his age, and a score of 9 indicating terminally ill patients. A score of 4 refers to a vulnerable person, not dependent on others for daily helps, but who have symptoms that limit the activities.

Statistics: Non-parametric data were expressed as median and interquartile range (IQR), categorical data as count and percentage. The comparison between groups was performed using Mann–Whitney test for nonparametric variables or Fisher exact test for categorical variables. The area under the curve of the receiver operating characteristic (ROC) was used to define the CFS cut-off value between frail versus non-frail patients respecting to favorable versus unfavorable 6-months outcome. A binary logistic regression analysis was used to evaluate the association between the variables and the patient’s outcome at 6-months, dichotomized as unfavorable (GOSE 1–3) vs. favorable (GOSE 4–8). Prism and SPSS (IBM SPSS Statistics for Windows, Version 22.0) were used for the analysis. A *p* value <0.05 was considered statistically significant.

## Results

During the study period 142 TBI patients were admitted to NICU, of whom 60 (42%) were at least 65 years old and 39 (65%) were males. A flow chart describing the patient’s inclusion process is shown in Supplementary Figure S1. Demographic characteristics, clinical and radiological parameters are presented in Table 1. The median age of this cohort was 76 (IQR 70–80), with a GCS at admission of 8 (IQR 6–11) and a GCS motor score of 5 (IQR 4–5). Twenty-four (40%) patients had at least one pupil not reacting to light. Absent/compressed basal cisterns and traumatic subarachnoid hemorrhage were present in 62 and 72% of patients, respectively, on admission CT scan, while midline shift was greater than 5 mm in 50% of cases. Associated comorbidities: neurologic diseases such as previous stroke, dementia, Parkinson’s disease, and seizures were present in 30% of patients; cardio-pulmonary diseases (i.e., chronic obstructive pulmonary disease or New York Heart Association functional classification ≥2 affected 50% of cases); antiplatelet and anticoagulative medications were taken by 38 and 20% of patients. Neurosurgical evacuation of a mass lesion was performed in 55% of patients (Table 1).

**Table 1:** Demographic characteristics, clinical and radiological parameters, surgical management and outcome of patients.

	All population (*n* = 60)	Not-frail (*n* = 37)	Vulnerable-frail (*n* = 23)	*p*-value
Age (years)	76 (70–80)	75 (69.5–80.5)	77 (72–79)	0.626
**Male**	39 (65)	26 (70)	13 (57)	0.404
Neurodegenerative disease	18 (30)	6 (16)	12 (52)	0.004
Cardio-respiratory disease	30 (50)	12 (32)	18 (78)	0.001
Antiplatelet therapy	23 (38)	12 (32)	11 (48)	0.281
Anticoagulant therapy	12 (20)	3 (8)	9 (39)	0.006
Prehospital parameters				
Hypotension	4 (7)	3 (8)	1 (4)	–
Hypoxia	6 (10)	5 (14)	1 (4)	0.391
ICU admission parameters				
GCS	8 (6–11)	8 (7–12)	6 (4–10)	0.199
GCS motor score	5 (4–5)	5 (4–5)	4 (2–5)	0.105
Fixed or dilated pupils	24 (40)	10 (27)	14 (61)	0.015
Surgery and CT scan characteristics				
Midline shift (1° CT scan)	2.1 (0–6.5)	0 (0–4.8)	3.8 (0–8.5)	0.122
Midline shift (worst CT scan)	5.1 (1.4–11.9)	4.4 (0–9.4)	8.5 (3.1–12.2)	0.235
Cisterns compressed or absent	37 (62)	21 (57)	16 (70)	0.416
tSAH	43 (72)	25 (68)	18 (78)	0.557
Lesion >25 ml	31 (52)	15 (41)	16 (70)	0.036
Surgery for mass lesion	33 (55)	18 (49)	15 (65)	0.287
Decompression	9 (15)	7 (19)	2 (9)	0.460
Outcome				
ICU mortality	24 (40)	7 (19)	17 (74)	<0.001
Hospital mortality	25 (42)	7 (19)	18 (78)	<0.001
GOSE at 6-months	1 (1–4)	3 (1–7)	1 (1–1)	<0.001
GOSE <4 at 6-months	40 (67)	19 (51)	21 (91)	0.002

Data are presented as count (%) or median (interquartile range). GCS, Glasgow Coma Scale; CT scan, computerized tomography scan; tSAH, traumatic subarachnoid hemorrhage; ICU, intensive care unit; GOSE, extended Glasgow Outcome Scale.

### Frailty

The median CFS was 3 (IQR 2–4). The distribution of CFS within the age intervals is shown in Supplementary Figure S2. The area under the ROC curve of CFS for prediction of mortality and GOSE at 6-months were 0.82 (*p* < 0.01) and 0.74 (*p* = 0.03), respectively. The best cut-off to discriminate between frail versus non-frail patients respecting to favorable versus unfavorable 6-months outcome was a CFS ≥ 4, with a specificity of 90% and a sensitivity of 53% (Supplementary Figure S3). Twentythree (38%) patients were classified as “vulnerable-frail” [CFS was 4 in 13 (22%) patients, 5 in 6 (10%) patients, 6 in 2 (3%) patients and 7 in 2 (3%) patients], while 37 (62%) patients were classified as “not-frail” [CFS was 1 in 2 (3%) patients, 2 in 13 (22%) patients and 3 in 22 (37%) patients] (Supplementary Figure S4). The 2 groups were similar in terms of age, GCS and CT characteristics such as status of the basal cisterns, midline shift and traumatic subarachnoid hemorrhage. Frail patients had a higher prevalence of fixed or dilated pupils and greater lesion occupying space compared to not-frail patients. The full comparison between frail and non-frail patients is shown in Table 1.

### Outcome

**Figure 1:**
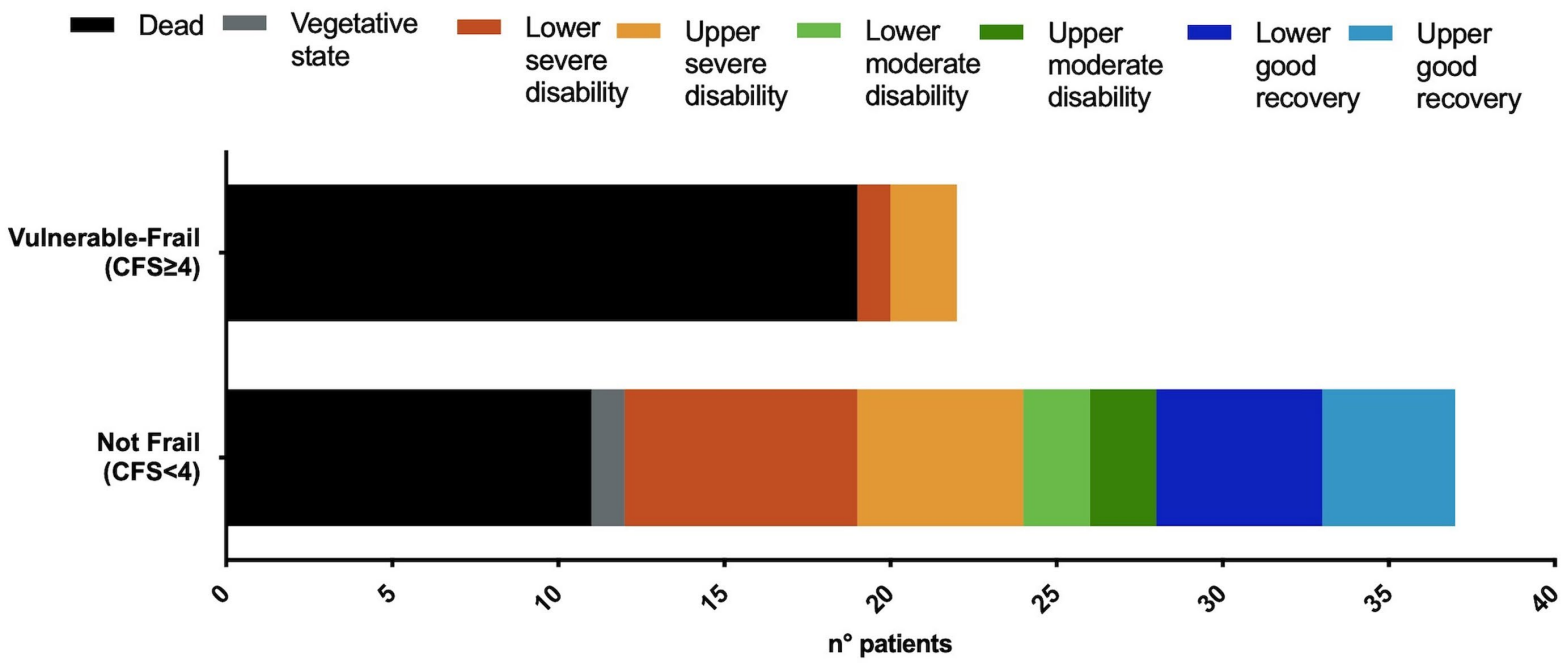
Distribution of the extended Glasgow Outcome Scale (GOSE) at 6-months, in vulnerable-frail and not-frail patients. CFS, Clinical Frailty Scale. Dead, GOSE 1; vegetative state, GOSE 2; lower severe disability, GOSE 3; upper severe disability, GOSE 4; lower moderate disability, GOSE 5; upper moderate disability, GOSE 6; lower good recovery, GOSE 7; upper good recovery, GOSE 8.

At 6 months after TBI death occurred in 31 (52%) patients, while unfavorable outcome in 40 (67%) patients. Vulnerable-frail patients showed 6-month mortality of 87% and unfavorable outcome of 91% that were significantly higher than those of non-frail patients, respectively 30% [OR and 95% CI: 15.7 (3.9–55.2), *p* < 0.001] and 51% [OR and 95% CI: 9.9 (2.1–46.3), *p* = 0.002] (Table 1). Figure 1 shows the distribution of GOSE at 6-months in the 2 groups of patients.

Univariate analysis showed that patients with unfavorable outcome were more frequently male and vulnerable-frail (CFS ≥ 4), had a higher prevalence of pre-existing neurodegenerative disease, pupillary abnormalities and lower GCS. They also had worst CT scan characteristics (greater shift at CT scan, higher prevalence of compressed of absent cisterns and traumatic subarachnoid hemorrhage). However only CFS ≥ 4 and the presence of traumatic subarachnoid hemorrhage remained associated with adverse outcome in the multivariate analysis, as shown in Table 2.

**Table 2:** Univariate and multivariate analysis for GOSE at 6-months.

	Univariate		Multivariate	
	OR (95% CI)	*p*-value	OR (95% CI)	*p*-value
Age	1.02 (0.93–1.13)	0.622		
**Male**	0.22 (0.04–0.77)	0.029*		
Neurodegenerative disease	6.0 (1.22–29.48)	0.027*	–	ns
Cardio-respiratory disease	1.35 (0.46–3.97)	0.584		
Antiplatelet therapy	0.90 (0.30–2.77)	0.851		
Anticoagulant therapy	1.64 (0.42–8.2)	0.496		
Vulnerable-Frail (CFS ≥ 4)	9.95 (2.03–48.64)	0.005*	7.84 (1.40–43.97)	0.019
Pre-hospital parameters
Hypotension	1.54 (0.15–15.83)	0.716		
Hypoxia	0.46 (0.08–2.52)	0.370		
ICU admission parameters
GCS	0.77 (0.63–0.93)	0.008*	–	ns
GCS motor score	0.76 (0.51–1.12)	0.169		
Fixed or dilated pupil	4.0 (1.14–14.08)	**0.031**		
CT scan characteristics				
Shift at 1° CT scan	1.08 (0.96–1.21)	0.187		
Shift at worst CT scan	1.11 (1.01–1-24)	**0.038**		
Cisterns compressed or absent	3.95 (1.28–12.27)	0.017*	-	ns
tSAH	4.71 (1.42–15.620)	0.011*	5.47 (1.13–26.58)	0.035
Lesion >25 ml	2.79 (0.91–8.50)	0.072		

*Variables with *p*-value < 0.03 were entered in multivariate analysis adjusted for sex. CSF, Clinical Frailty Scale; GCS, Glasgow Coma Scale; CT scan, computerized tomography scan; tSAH, traumatic subarachnoid hemorrhage. Bold value indicates statistically significant (*p* < 0.05).

## Discussion

In our study we observed that 38% of older people with TBI were vulnerable-frail and that CFS ≥ 4 was associated with 6 month-outcome, suggesting that the pre-injury functional status could represent an additional indicator to stratify patients’ severity and predict the outcome.

During the past years, frailty has become a major topic of research in several fields of medicine including critical care, and has been associated with complications, prolonged length of hospital stay, functional decline, reduced quality of life and increased mortality (12). Galimberti et al. (8) evaluated a FI in 2993 TBI patients and documented an association between the higher FI and unfavorable 6-month outcome regardless of patients’ age. They used a FI based on 30 items, using a deficit accumulation model based on comorbidities, pharmacological therapy, and laboratories values within the first 24 h after admission, but without information about pre-injury nutritional assessment, physical activity, social life, cognition, psychological condition etc., that are all relevant aspects in frailty evaluation. Their patients are not fully comparable with ours, since the median age of patients in Galimberti’s study was 51, the median admission GCS was 14, and older patients were only 29% of the population. Only 18% of their cohort had a FI > 0.2, the cut-off used to define frailty. In our study, we focused only on older patients, and 38% of patients were vulnerable/frail.

More recently Tang et al. retrospectively evaluated 691,821 patients from the National Trauma Data Bank with traumatic intracranial hemorrhage investigating the association between frailty and outcome. Patients’ age was 58 ± 21, 75% of the patients were admitted for mild TBI, 25% for moderate–severe TBI. Frailty was quantified using the validated modified 5-item FI (mFI-5) metric (range = 0–5) that was calculated based on the presence of congestive heart failure, diabetes mellitus, chronic obstructive pulmonary disease (COPD), functionally dependent status, and hypertension, with each comorbidity contributing 1 point. Frailty was defined as index ≥2 and increased during the 10 years observation period from 8 to 22% of the patients. The authors observed that frailty, was associated with increased complications, ICU-hospital length of stay and mortality (9). However, the 5-item FI is very simplified to capture the multidimensional nature of frailty, it evaluates an association with comorbidities, and only 1 item is related to the functional status of the patient. Despite these differences, all studies share the common message that frailty is associated with adverse outcomes after TBI.

To date, there is no consensus about the best method to define frailty and there aren’t universally accepted assessment tools, reference standards, clinical criteria, or biological markers. The CFS has been found to be associated with a variety of patient characteristics and clinical outcomes, particularly in acute care. In the recent VIP2 study evaluating 3,920 patients older than 80 years old and admitted to ICU, the CFS was independently associated with mortality at 30 days. However, only less than 5% of the patients had TBI (13). Since the CFS combines clinical evaluation with objective measurements and can be easily obtained, it is considered one of the most promising and practical ways of screening frailty (6). Most of the studies performed in acute care setting have dichotomized the CSF and have found an association between CFS > 4 and adverse outcome. However, none of these studies specifically focused on older people with TBI. We dichotomized vulnerable/frail versus non frail patients based on our ROC curve that identified a cut-off of CFS ≥ 4 for distinguishing favorable vs. unfavorable outcome at 6-months, suggesting that in the setting of TBI in the older people also vulnerable patients are at risk of adverse outcome. We chose this cut-off with high specificity at the expense of low sensitivity (90 and 53%, respectively), because we believe that the identification of the frail patients, with a very low rate of false positive, will avoid the inappropriate withdrawn of therapies in older people who may even benefit from aggressive treatments.

In our population prevalence of pre-existing neurodegenerative and cardiorespiratory disease and anticoagulant medications were significantly higher in patients included in the frail group than the in non-frail group. Frail and non-frail patients were similar in terms of admission GCS, pre-hospital hypotension and hypoxia; in contrast, frail patients had a higher prevalence of fixed or dilated pupils and greater lesion occupying space compared to not-frail patients. Other CT scan parameters and occurrence of surgery did not differ between groups. In the univariate analysis, we found an association between adverse outcome and low GCS, presence of pupillary abnormalities, basal cisterns compression, midline shift, tSAH and frailty, while in multivariate analysis only tSAH and CFS ≥ 4 remained significantly associated with GOSE at 6-months. We explain the lack of association between the occurrence of hypotension/hypoxia and adverse outcome with the fact that they occurred only in a minority of patients; in addition, the fact that in multivariate analysis, significance between GCS, pupillary abnormality, basal cisterns status and outcome was lost, could be explained by the reduced sample-size of our population.

The mechanisms underlying the association between frailty and adverse outcome after TBI have not been elucidated. The existing data exclude that frail patients sustained a more severe TBI, or had a more complicated course because of neurologic secondary insults such as intracranial hypertension, seizures etc. Potential mechanisms are dependent on a reduced functional reserve, proinflammatory status, immunosenescence, limiting the capability to survive to the common complications associated with hospitalization and bed rest during the acute phase such as infections and organs failure that might occur. Another explanation is a reduced reserve for recovery during the rehabilitation phase associated with pre-existing neurodegeneration, cognitive decline, depression, sarcopenia and neuromuscular weakness (14).

Our findings suggest clinical and research implications. Frailty should become a standard clinical evaluation tool for older people at hospital admission after any form of acute brain damage, adding complementary information to the patients’ age to better predict the course of TBI, ischemic and hemorrhagic stroke. Frailty should also be incorporated into prognostic models to increase the accuracy of outcome prediction and could be a further aspect to consider in conditions of limited resources to avoid futile treatments in patients with acute brain damage and no chance of recovery.

Some of the aspects of frailty are potentially reversible, therefore proactive individualized interventions should be given to vulnerable older people to prevent injuries, to better tolerate the complications of hospitalization, and to maximize the chance of recovery during the rehabilitation phase. Finally, more work needs to be done to evaluate how to best assess frailty in patients with acute brain damage, that are prone to physical and cognitive deficits.

Our study has several limitations: it is a single center observational study, and only 60 patients were evaluated, therefore it should be seen as a preliminary report that requires further confirmation.

## Data availability statement

The raw data supporting the conclusions of this article will be made available by the authors, without undue reservation.

## Ethics statement

The studies involving human participants were reviewed and approved by Ethics committee of Papa Giovanni XXIII Hospital. The patients/participants provided their written informed consent to participate in this study.

## Author contributions

LZ and LL: conceptualization, data curation, formal analysis, investigation, methodology, writing, and review and editing. SA: data curation, investigation, and writing. RZ: data curation, formal analysis, and review and editing. FM: data curation. PG, FB, and LFL: review and editing and supervision. FB and LFL: funding acquisition. All authors read and approved the final manuscript.

## Funding

This work has been supported by Brembo SpA (Curno, Bergamo, Italy). The funder was not involved in the study design, collection, analysis, interpretation of data, the writing of this article or the decision to submit it for publication.

## Conflict of interest

The authors declare that the research was conducted in the absence of any commercial or financial relationships that could be construed as a potential conflict of interest.

## Publisher’s note

All claims expressed in this article are solely those of the authors and do not necessarily represent those of their affiliated organizations, or those of the publisher, the editors and the reviewers. Any product that may be evaluated in this article, or claim that may be made by its manufacturer, is not guaranteed or endorsed by the publisher.

## Abbreviations

GCS, Glasgow Coma Scale
FI, Frailty Index
CFS, Clinical Frailty Scale
extended Glasgow Outcome Scale
