# Supplementary figures and images for: Clinical frailty scale as a predictor of outcome in elderly patients affected by moderate or severe traumatic brain injury
Source: Front Neurol. 2023 Apr 6;14:1021020. doi: 10.3389/fneur.2023.1021020 (PMC10116041; doi:10.3389/fneur.2023.1021020)

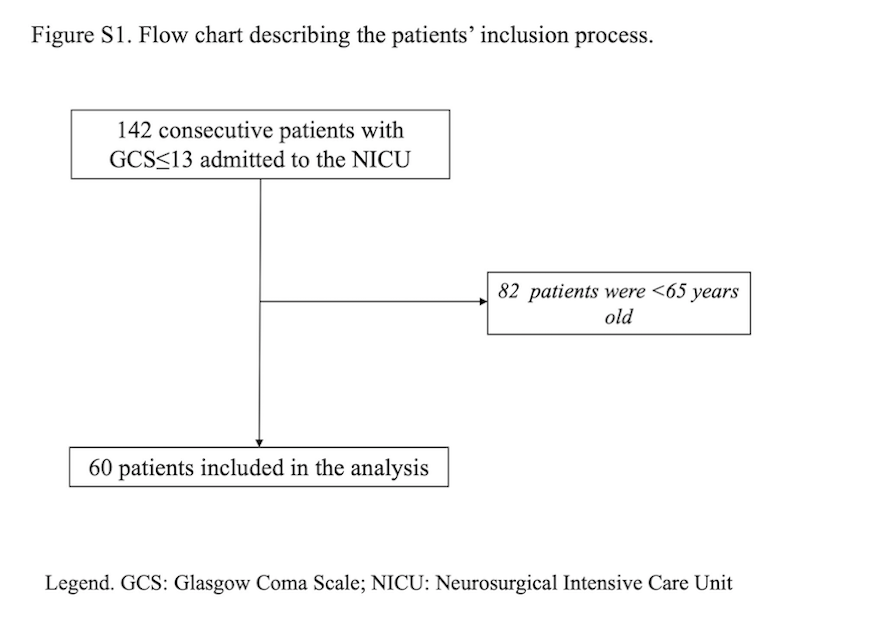

Supplement: Supplementary file 1 [file Image_1.tiff]

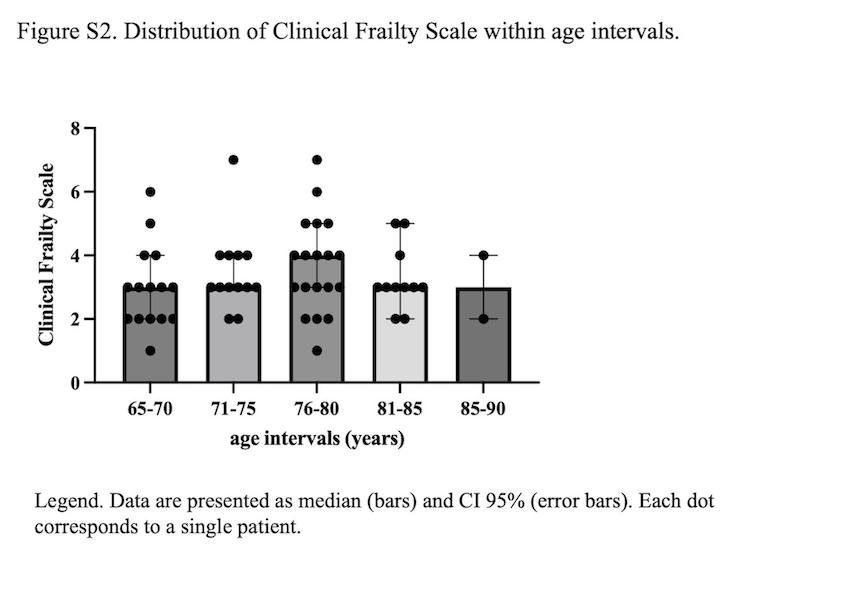

Supplement: Supplementary file 2 [file Image_2.tiff]

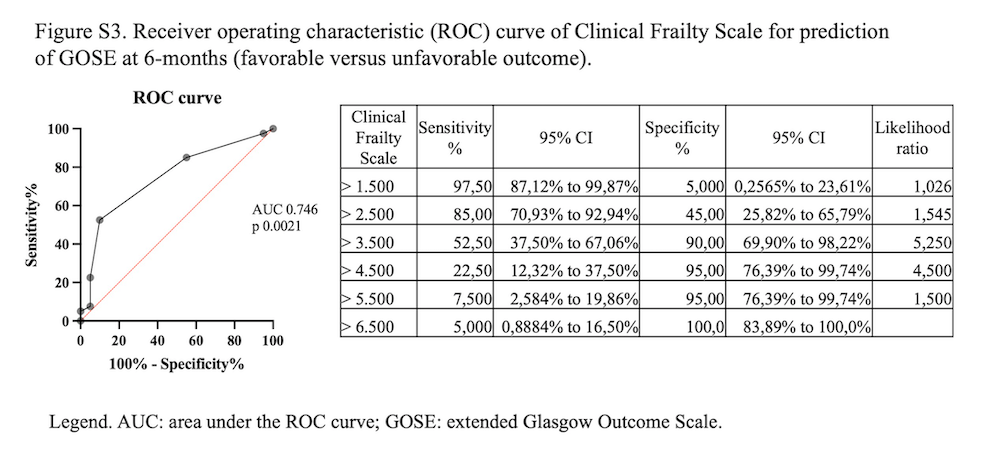

Supplement: Supplementary file 3 [file Image_3.tiff]

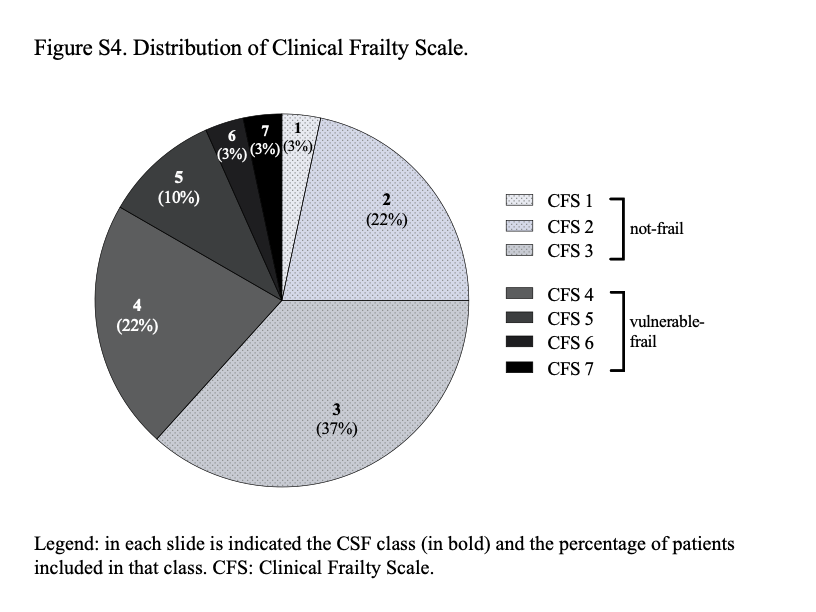

Supplement: Supplementary file 4 [file Image_4.tiff]
